# Humanized Mouse Models for the Study of Hepatitis C and Host Interactions

**DOI:** 10.3390/cells8060604

**Published:** 2019-06-17

**Authors:** Kylie Su Mei Yong, Zhisheng Her, Qingfeng Chen

**Affiliations:** 1 Institute of Molecular and Cell Biology, Agency for Science, Technology and Research (A*STAR), Proteos, Singapore 138673, Singapore; yongsmk@imcb.a-star.edu.sg (K.S.M.Y.); zsher@imcb.a-star.edu.sg (Z.H.); 2 Department of Physiology, Yong Loo Lin School of Medicine, National University of Singapore, Singapore 117545, Singapore; 3 Key Laboratory for Major Obstetric Diseases of Guangdong Province, The Third Affiliated Hospital of Guangzhou Medical University, Guangzhou 510150, China

**Keywords:** humanized mice, hepatitis C virus, liver, hepatotropic disease, steatosis, cirrhosis, hepatocellular carcinoma

## Abstract

Hepatitis C virus (HCV) infection is commonly attributed as a major cause of chronic hepatotropic diseases, such as, steatosis, cirrhosis and hepatocellular carcinoma. As HCV infects only humans and primates, its narrow host tropism hampers in vivo studies of HCV-mammalian host interactions and the development of effective therapeutics and vaccines. In this context, we will focus our discussion on humanized mice in HCV research. Here, these humanized mice are defined as animal models that encompass either only human hepatocytes or both human liver and immune cells. Aspects related to immunopathogenesis, anti-viral interventions, drug testing and perspectives of these models for future HCV research will be discussed.

## 1. Introduction

First identified in 1989, hepatitis C virus (HCV) is an enveloped, positive-sense, single-stranded ribonucleic acid (RNA) virus belonging to the genus Hepacivirus of family Flaviviridae [1,2]. It is hepatotropic and a leading cause of liver disease including acute hepatitis, chronic hepatitis, cirrhosis and hepatocellular carcinoma. The global prevalence of HCV infection is estimated at 3%, with 170 million chronically infected individuals [3,4,5]. Infection is transmitted through blood and most frequently occurs by perinatal transmission, unsafe invasive healthcare procedures and improper injection practices [6,7].

To date, there are 7 major HCV genotypes (1–7) [8]. Despite their different geographical distributions, there is about 30% heterogeneity in the viral genomic sequence between each genotype [9,10]. For example, genotype 4 is found in Egypt, genotype 5 in South Africa, genotype 7 in Democratic Republic of Congo (DRC) and genotypes 1a, 1b, 2 and 3 within United States of America [9,11]. On the other hand, having one of the highest global incidences of HCV, the predominant genotypes in Asia are genotypes 1 (Singapore, Indonesia and Philippines), 3 (Malaysia and Thailand) and 6 (Vietnam, Myanmar and Laos) [12].

A spectrum of in vitro and in vivo models including; cell culture, tree shrew, zebrafish, chimpanzee and viral protein transgenic mouse models have been used to study HCV [4]. Even though these models have advanced parts of HCV research, the inability to fully recapitulate relevant clinical features as observed in human patients, create a need for improved animal models to enable the effective study of HCV immunopathogenesis and evaluation of new therapeutics and prophylactic vaccines [13].

Hepatitis C virus exhibits a narrow host tropism, infecting the hepatocytes of a limited range of species, particularly human and chimpanzee [14,15]. Being genetically similar to humans, the chimpanzee has been instrumental in the study of HCV infection. Its contributions to research include the identification of HCV, understanding HCV immunopathogenesis, validation of molecular tools for drug discovery and evaluation of drug safety/efficacy [16]. The accumulation of scientific discoveries, has translated into several Food and Drug Administration (FDA)-approved antivirals [17]. While the chimpanzee models have provided valuable insights to HCV research, the use of these models is limited due to the lack of human cells/immune system, ethics and economic reasons [16].

Existing limitations and the need to further our understanding of HCV viral immunopathogenesis and treatments; have led scientists to establish HCV infected humanized mouse models [18,19,20,21]. In this context, humanized mice are defined as mice engrafted with only human hepatocytes or with human immune system and hepatocytes. These small animal models allow in vivo HCV infection as per clinical settings, therefore enabling the analysis of human-specific host immune responses [18,19,20,21]. In this review, we provide an overview of the currently available humanized mouse models that have proven valuable for the study of HCV and discuss their main benefits and weaknesses.

## 2. Life Cycle of HCV

The HCV life cycle is only partially understood as there is a complex network of cell surface molecules involved in mediating viral entry, hence making it challenging to establish a reliable in vitro model of replication [22]. It has been shown that there are seven steps to the lifecycle of HCV, namely; attachment, entry, uncoating, translation, replication, assembly and maturation. Viral particles of HCV circulate the blood either as free particles or are surrounded by low-density lipoproteins from the host. These HCV virions then enter the cells via clathrin-mediated endocytosis by sequentially binding various receptor molecules of the target cell membrane [22,23,24].

Low pH of the endosome triggers fusion of cellular and viral membranes, in turn causing the capsid to be disorganized. Upon disruption of the viral capsid in the endocytic compartment, single-stranded RNA genome is uncoated and released into the cytoplasm. Following this, the RNA genome is translated at the rough endoplasmic reticulum (ER) to form a single polyprotein precursor which is cleaved by cellular and viral proteases into single proteins. Products from this process include, structural core and envelope glycoproteins E1 and E2; non-structural proteins important for viral assembly and release, p7 viroporin and non-structural protein 2 (NS2); protease complex, NS3 and NS4A; membrane-associated protein which mediates interactions between virus and host, NS4B; Zinc-binding and proline-rich hydrophilic phosphoprotein essential for replication of HCV RNA, NS5A and a RNA polymerase, NS5B [22,23,24].

Through a minus-strand replicative intermediate, an array of host factors and non-structural proteins form a replication complex which makes multiple copies of HCV RNA genome. Virions undergo maturation and are enveloped by endogenous lipoproteins as they are being assembled in an ER-derived compartment and finally released through exocytosis via a Golgi-dependent secretory pathway or transmitted to other cells by a cell-free mechanism [22,23,24].

Among the most frequently utilized methods to study in vitro replication of HCV, cell culture HCV (HCVcc) and HCV trans-complemented particles (HCVTCP) are most widely applied. Application of these cell lines has not only allowed the identification of HCV entry factors but also established the virion structure of HCV, determined its biochemical properties and evaluated relevant therapeutics. However, all in vitro methods use Huh-7 cells. Even though these cells are permissive to HCV replication, it has different mechanisms, locations of HCV receptors and absent polarity as compared to primary hepatocytes [22,23,24]. As a result, the life cycle of HCV is not accurately reproduced in this model, hence making humanized mice an attractive platform to further understand the life cycle of HCV.

## 3. Immunopathogenesis of HCV

Once hepatocytes are infected with HCV, the immune system is activated to clear the virus [25,26]. To do this, a cascade of immunological events is triggered, dendritic cells (DC), hepatic stellate cells and Kupffer cells secrete cytokines (MIP-1α, IL-12, IL-15 and IL-18) to recruit NK cells, which produce IFNγ in the liver [27,28]. Type I and III interferons produced, then activates sinusoidal endothelial cells to secrete chemokines (CXCL10, MIG), which attracts T cells for viral clearance [29].

However, the highly mutable HCV is capable of evading host immune system to develop chronic infection. It achieves this through a number of ways, first via the disruption of DCs. It has been shown that HCV can increase levels of indoleamine-2, 3, -deoxygenase to dysregulate DC maturation and antigen-presenting functions [30]. Induce plasmacytoid dendritic cell (pDC) apoptosis and disrupt proteasomal subunits of DC to affect their phagocytic functions [31]. Unresponsiveness to chemokine CCL21 also impairs DC migration to lymphoid tissues and downregulates HCV-sensing toll-like receptors (TLRs) as well as critical adaptor molecules (TRIF, TRAF6) [32]. Impaired DC function, early in HCV infection results in low levels of NK cell maturation and an immunosuppressive regulatory T-cell phenotype due to defective priming of CD4^+^ and CD8^+^ T cells [33,34,35].

Second, even though it has been shown that there is no difference in the cytotoxicity of natural killer (NK) cells between groups of individuals including HCV infected, recovered patients and healthy donors [36,37,38,39]. Researchers have demonstrated that NK cells may be inhibited by ways including but not limited to, reduction in NK cell expression of microRNA-155, which upregulates inhibitory receptor Tim-3; disrupted NK cell activation receptor expression and core-induced stabilization of human leukocyte antigen E (HLA-E) [36,37,38,39,40,41].

Third, assisting HCV in evading the immune system also include CD56^+^ natural killer T cells (NKT), which are found to be at low levels in acute HCV infection [42]. Additionally, in chronic infection, naïve antigen-specific CD8^+^ T cells have been shown to be primed by NKT cells to produce an immunosuppressive environment, further enabling HCV to evade the immune system [43]. 

Lastly, early adaptive immune responses are crucial in determining the acute or chronic outcome of HCV infection. About 30% of infected individuals are able to eliminate virus-infected cells via strong and sustained responses by CD8^+^ T cells and CD4^+^ T cells [44,45,46,47,48]. A defined role for B cells in HCV infection has not been dissected and current results are controversial [49]. In chronic infection, the inability of T cells to control HCV infection could be due to a range of reasons, such as, defective priming of T-cells by DC, abnormal T cell priming by intrahepatic antigen-presenting cells, T cell anergy and high expression levels of viral antigens in hepatocytes that causes elevated levels of regulatory T cell (Treg) subsets, which in turn creates an immunosuppressive immune environment [28,50,51,52].

## 4. The Role of HCV in the Progression of Liver-Associated Diseases

In acute hepatitis infection, components within the extracellular matrix, including glycoproteins and proteoglycans are synthesized in a tridimensional network to limit inflammatory reactions during the early phases of infection [53]. However, when infected individuals are unable to clear the virus, chronic HCV infection occurs. During this state, activated hepatic stellate cells acquire a myofibroblastic phenotype which allows it to attract leukocytes, proliferate and produce extracellular matrix proteins and collagen, eventually resulting in hepatic fibrogenesis where these components are deposited in the liver and eventually damage the organ [54].

The transition from liver fibrogenesis and cirrhosis involves inflammatory factors, vascular occlusion and angiogenesis. The activity of hepatic stellate cells is mainly regulated by important elements such as, transforming growth factor β (TGF-β), chemokines and adipokines [54]. Similar to fibrogenesis, both the formation of angiogenesis and cirrhosis are mediated by extracellular matrix remodeling and the activation of growth factors, including but not exclusive of TGF-β, vascular endothelial growth factor (VEGF), and platelet-derived growth factor (PDGF), and increased gene expression of adhesion molecules [55,56].

Hepatocellular carcinoma (HCC) is the most common type of primary liver malignancy and the fourth leading cause of cancer-related deaths globally [57]. In most cases, HCV-associated hepatocarcinogenesis is preceded and driven by hepatic inflammation, oxidative stress and cellular deoxyribonucleic acid (DNA) damage [54,58]. All of which, is progressively caused by hepatic fibrogenesis and cirrhosis [53,59]. Once infected, hepatitis C virus does not integrate into the host’s genome. Instead, it remains as an episome in the endoplasmic reticulum (ER), encoding for survival and growth proteins, which induces the expansion of infected hepatocytes [60,61]. Proliferation of infected hepatocyte activates signaling pathways, such as Wnt/β-catenin and MAPK/ERK, which results in mutations implicated during the transition of chronic viral hepatitis to HCC [62,63,64].

## 5. Types of Humanized Mice for HCV Studies

### 5.1. Humanized Mouse Models with Only Human Hepatocytes

As HCV has a narrow host tropism, there is a lack of in vivo models that can recapitulate clinical settings, therefore limiting the development of effective vaccines and treatments [65]. Even though non-human primate animal models have contributed significantly to HCV research, ethical, financial and a lack of human immune system have restricted their use in recent years [16]. 

This limitation raised enormous demands for a small, easy to manipulate and cost-effective model that is able to recapitulate clinical scenarios [14]. These include different types of humanized mice, of which immunodeficient mice engrafted with either only human hepatocytes or both human immune system and hepatocytes [18,19,21,66,67,68,69,70,71,72,73,74,75,76]. These models allow the study of HCV infection and discovery of potential treatments. Here, we review humanized mouse models currently available for HCV research and have included important information, such as details of their study and advantages as well as limitations for each model.

#### 5.1.1. Alb-uPA/SCID

The Alb-uPA/SCID model was designed to investigate neonatal bleeding disorders [77]. The over-expression of murine urokinase-type plasminogen activator (uPA) with an albumin promoter resulted in an increase in plasma uPA levels, severe hemorrhagic events, accelerated hepatocytes and neonatal death [77,78]. However, in newborn mice, scientists observed that the elevated uPA levels gradually decreased to normal levels through random somatic deletions of the uPA transgene within hepatocytes and therefore abolished transgene expression. These transgenic uPA deficient cells exhibited significant replicative advantage, capable of selective proliferation and complete liver regeneration [78] (Table 1).

In a study to investigate the replicative capacity of adult mouse hepatocytes into newborn Alb-uPA/SCID mice, these mature hepatocytes replaced up to 80% of the hepatocytes in recipient liver and restored liver deficiency [78,79]. By transplanting normal human hepatocytes into Alb-uPA/SCID mice, in vivo human HCV infection was demonstrated for the first time by Mercer et al. [80]. These chimeric Alb-uPA/SCID mice were inoculated with HCV infected human serum and developed prolonged HCV infections of up to 35 weeks with high viral titers. The human liver Alb-uPA/SCID mouse models have proven valuable for its contributions to understanding the basic biology of HCV, evaluation of antiviral therapies and neutralizing antibodies [81,82,83,84,85,86,87,88,89].

Yet, limitations of this model include, high neonatal mortality rate, susceptibility to kidney disorders, small body size and weight, limited time window for transplantation, breeding difficulties and the inability to expand engrafted human hepatocytes due to the spontaneous genotype reversion in recipient mouse hepatocytes, that effectively outcompete the transplanted human cells during liver repopulation [4,90]. 

#### 5.1.2. cDNA-uPA/SCID

To address existing issues, variants of uPA mice, such as hemizygous cDNA-uPA/SCID was established and successfully infected with HCV in vivo [91]. This strain was generated using embryonic stem cell techniques and attained appropriate levels of uPA expression. As compared to the original strain, the liver of this model was not detrimentally damaged, experienced fewer kidney disorders, and had higher body weight and longer survival rates. However, even though HCV viremia was significantly higher in this model, it was unable to maintain viremia for longer than 8 weeks [70].

#### 5.1.3. MUP-uPA SCID

The other variant, MUP-uPA SCID/Bg model was constructed by backcrossing transgenic mice carrying the uPA gene driven by MUP promoter onto a SCID/Beige background [92]. As compared to the original strain, the MUP-uPA SCID/Bg mouse is healthier, has a longer time window of up to 1 year of age for hepatocyte transplantation and is susceptible to infection with HCV genotype 1-6 [71].

#### 5.1.4. uPA/NOG

Constructed on severely immunodeficient non-obese diabetic (NOD)/Shi-scid/IL-2Rγnull (NOG) mice, the third variant is the uPA/NOG mouse model [93]. This model offers several advantages over classical Alb-uPA/SCID mice, enabling, minimal neonatal lethality, increased breeding efficiency, improved recipient survival, simplified surgical manipulation and higher xenogeneic cell engraftment. At present, HCV infection has not been reported in uPA/NOG mice.

#### 5.1.5. Fah^−/−^/Rag2^−/−^/Il2rg^−/−^ (FRG)

The FRG strain of mice is a triple knockout mouse model [73]. Deletion of fumarylacetoacetate (Fah), a tyrosine catabolic enzyme results in the accumulation of hepatotoxic metabolites such as Fah and maleylacetoacetate which induces liver damage and is lethal [94] (Table 2). Oral administration of 2-(2-nitro-4-trifluoro-methylbenzoyl)1,3-cyclohexedione (NTBC) blocks the enzyme hydroxyphenylpyruvate dioxygenase which prevents the accumulation of hepatotoxic metabolites, liver damage and maintains FRG mice in a healthy state [94]. Upon NTBC withdrawal, mouse liver can be repopulated with human hepatocytes [73,94].

The FRG model is advantageous to classical Alb-uPA/SCID mice in a range of ways including, that it has a higher rate of chimerism, is easy to breed and there is absolutely no spontaneous transgene revision, renal disorders and limitations in the time frame for transplantation as liver repopulation is controlled by NTBC withdrawal [74].

An absence of spontaneous transgene reversion in FRG enables serial transplantation of human hepatocytes as the Fah deficient mouse hepatocytes are unable to compete with transplanted human cells during liver repopulation. Because of this, an infinite number of hepatocytes from a single donor can be produced via serial transplantation over a few generations of mice, hence making it a cost-effective model, attractive for large scale studies requiring human hepatocytes or mice.

It has been reported that a high rate of liver chimerism of up to 95% human hepatocytes in FRG mice is generated by high transplantation dose of human hepatocytes. These mice were successfully infected with 4 HCV genotypes and were responsive to antiviral and neutralizing antibodies [74,95,96]. Improvements to FRG models are constantly being developed by adding human oncostatin-M to enhance human hepatoblastoma repopulation in recipient mouse liver by 5-100-fold [96]. These syngeneic liver and immune system mice are reconstituted with functional human T and B lymphocytes, monocytes and NK cells, which are able to support HCV infection, hence making it an ideal model for the study of HCV infection in the liver.

#### 5.1.6. TK-NOG

In 2011, a new mouse model expressing a transgene, herpes simplex virus type 1 thymidine kinase (HSVtk), within the liver of immunodeficient NOG mice (TK-NOG) [97] was created. Mouse liver cells diminished after the exposure of ganciclovir (GCV) and human hepatocytes were stably maintained without exogenous drugs. It has been shown that serum alanine aminotransferase (ALT) levels are increased in TK-NOG mice after the HCV infection induced by GCV treatment, rendering these mice useful for the study of HCV virology [76,97]. However, drawback of this model includes a lack of human immune system, absence of liver disease post-infection, low body weight and high rates of renal disorders and mortality [76,97]. 

### 5.2. Humanized Mouse Models with Human Immune System and Hepatocytes

Human liver chimeric mice have provided valuable insights into HCV infection, as well as the evaluation of antiviral treatments. However, a major limitation includes the lack of a functional human immune system, which impedes the understanding of human-specific immune responses during HCV immunopathogenesis and novel therapeutics/vaccines [67,98].

Histopathological features associated with chronically infected HCV patients, such as hepatic inflammation, fibrosis, cirrhosis and HCC has not been reported in human liver chimeric mice. This indicates that the human immune system plays a key role in disease progression. Therefore, humanized mouse models with both human hepatocytes and immune system should be used for HCV research.

#### 5.2.1. AFC8-hu HSC/Hep

Driven on an albumin promoter (AFC8), the AFC8-hu HSC/Hep strain of transgenic mouse expresses FK506 binding protein (FKBP) and caspase 8 [66,67]. To induce liver cell death, recipient mouse is administered AP20187, a synthetic drug that induces dimerization of active caspase 8, expressed specifically in mouse liver cells [66,67].

To study HCV pathogenesis, human CD34^+^ hematopoietic stem cells (HSC) and human hepatocyte progenitors were co-transplanted into Balb/C-Rag2^−/−^-γC^−/−^ mice and infected with HCV isolates [66]. Infected mice demonstrated elevated levels of ALT, pDC, NK cells, macrophages, T cells, liver inflammation and fibrosis [66,67].

Limitations of this model include, low human liver chimerism of 15%, undetectable HCV viremia in the blood, absence of HCV-specific antibodies and hepatitis C virus genomic RNA in the liver, is detectable in only half of infected mice, after 1-4-month post-infection [66,67] (Table 3).

#### 5.2.2. NSG-DRB*0101

Even though current humanized mouse models with both human hepatocytes and immune system are crucial for modelling diseases and testing drugs, it has been reported to have some functional deficiencies. The NSG-DRB*0101 mouse model was developed to determine if the inclusion of human leukocyte antigen (HLA) would improve the development of functional human T and B cells [99,100,101,102,103].

Expression of HLA in humanized mice allowed the development of a partially functional adaptive human immune system during viral infection and the generation of HLA-A2 restricted virus-specific T cells. However, drawbacks in this model include difficulty in sourcing HSC with the same HLA type, minute levels of human NK cells, defective recruitment and homing of human immune cells, and restricted inter-species cross-reactivity of cytokines and chemokines produced during infection [68].

#### 5.2.3. HIL Mice

A humanized mouse model with both human hepatocytes and immune system (HIL mice) was established by intrahepatically injecting mice with human fetal liver cells [18,104,105,106]. Unlike other chimeric liver mouse models, these mice do not require additional drug treatments or transgene modifications [18,104]. 

Similar to clinical scenarios, these mice developed liver inflammation, upregulated human cytokines and developed fibrosis [18,19,20,21]. Long-term effects of HCV in HIL mice were monitored for up to 28 weeks, where infected mice demonstrated higher incidences of fibrosis. Immune profile analysis of HCV infected mice showed elevated numbers of T cells and monocytes/macrophages in granulomatous inflammation [18,19,20,21] (Table 4).

In addition, at 28 weeks’ post-infection, human proinflammatory cytokines such as interferon gamma (IFN-γ), monocyte chemoattractant protein 1 (MCP-1) and interleukin 18 (IL-18) were significantly increased in the plasma of HIL mice [19]. When treated with pegylated-interferon-α-2A (PEG-IFNα-2A), progression of HCV liver pathogenesis was blocked by PEG-IFNα-2A, demonstrating that HIL mice are able to reproduce HCV infection and immunopathogenesis [107,108,109,110,111,112]. The main drawback of this model is the low levels of B cells detected as compared to clinical settings [19]. 

## 6. Hepatitis C Treatment

Humanized mouse models with only human hepatocytes have been crucial in initial therapeutic tests. However, due to the lack of a functional human immune system, the study of in vivo drug interactions and vaccine development has been precluded [113,114]. On the other hand, humanized mouse models with both human immune system and hepatocytes are able to efficiently recapitulate immune-mediated events in HCV and are essential in developing novel vaccines and treatments.

Depending on the genotype of HCV, different combinations of therapeutics including interferon (IFN), direct acting antiviral (DAA) and ribavirin (RBV) are usually prescribed [115,116,117,118]. Some obstacles faced in current therapies include factors such as, patients being resistant to existing treatments, poor tolerance to side effects which prevent individuals from completing therapy, limited efficacy, high cost and emergence of drug-resistant viral variants [119,120]. For these reasons, constant research and development is needed to develop effective treatment and vaccine options. In this review, some antivirals such as Claudin-1 (CLDN1), Interferon-λ, NA808, direct acting antivirals (DAA) and PEG-IFNα-2A will be discussed.

### 6.1. Claudin-1 Antibody

As a tight junction protein, functions of CLDN1 include regulation of HCV entry and transmission from cell to cell [121]. Claudin-1 antibody functions by eliminating HCV viral activity through inhibition of HCV cell entry. When Alb-uPA/SCID mice were administered CLDN1, virus-mediated signaling pathways were induced and persistent HCV infection was cleared, demonstrating effectiveness of the treatment [95,111] (Table 5).

### 6.2. Interferon-λ

Interferon is a cytokine that is essential in mediating the innate immune system and is the first response against HCV viral infection. The innate immunity is crucial for initial defense against viral pathogens; therefore, antiviral drugs that can activate innate immune response are ideal candidates for HCV treatment [122,123,124].

### 6.3. NA808

An alternative method of activating the innate immune system is through a complex, cationic liposome (LIC) and synthetic double-stranded RNA analog, polyinosinic-polycytidylic acid (Poly I:C) (LIC-pIC). Activation of this complex induces IFN-λ mediated antiviral response of HCV-infected human hepatocytes and is a potential for overcoming resistance to therapies across different HCV genotypes [124,125].

Katsume et al. [126] identified a novel class of serine palmitoyltransferase (SPT) inhibitor, known as NA808. Derived from fungal metabolites, this SPT inhibitor prevents the synthesis of sphingolipid, in turn disrupting HCV replication complex, hence inhibiting HCV replication.

### 6.4. DAA

Studies have shown that even in the absence of immunomodulatory IFN, direct acting antivirals (DAA) are able to activate the innate immune system. Administration of DAA suppresses HCV expression and significantly reduces levels of chemokines [96]. Post-DAA treatment, there is an increase in protein expression of Cardif and IFITM1, suggesting that DAA are able to block HCV expansion in hepatic cells [96,127].

Currently, an array of DAA is being developed as novel strategies to target HCV [11]. These drugs have been shown to provide shorter treatment times, higher cure rates and reduced side effects. The main classes of DAA are NS3/4 protease inhibitors (PIs), NS5A inhibitor, Nucleoside and nucleotide NS5B polymerase inhibitors. These drugs directly target viral proteins halt HCV replication in host cells. In clinics, treatments for HCV infected patients include combinations of DAA. Two significant groups of DAA are widely used, Boceprevir and Telaprevir blocks NS3/4A serine protease and inhibits crucial proteins for viral cycle, while NS5A inhibits protease to halt HCV replication [128,129,130,131].

### 6.5. PEG-IFNα-2A

Human PEG-IFNα-2A is often used in clinical treatment of HCV and has a specific role in inhibiting HCV replication and regressing HCV-associated disease progression. It has been shown that HIL mice are able to reproduce HCV infection, immunopathogenesis and drug response as per clinical settings. When tested with PEG-IFNα-2A, progression of HCV liver pathogenesis was halted. In addition, it was observed that serum ALT levels decreased and mice were protected from liver damage and fibrosis [18,132].

## 7. HCV Vaccines

Although current treatments have improved the cure rates of HCV, existing limitations that prevent complete disease eradication include; high cost of therapy, limited worldwide accessibility, potential development of drug resistance and an inability to ameliorate long-term effects of chronic infection [4]. Therefore, to relive a worldwide burden of the disease, it is crucial to develop an effective vaccine to prevent the transmission of virus and liver damage [133].

Over the years, a range of adjuvants, vectors and vaccination regimens have been tested [4]. At present, two ways are being used to design HCV vaccines. First, through inducing an antibody response that targets the exterior surface of viruses [134]. However, as HCV is highly variable among strains and mutates quickly, this method is extremely challenging. Second, generate broadly neutralizing antibodies to induce viral inactivity and third, stimulate broad T cell responses to clear infected hepatocytes [134,135,136,137].

The task of developing a vaccine against HCV is an extremely challenging process. Humanized mouse models can be readily used to better understand the immunopathogenesis of HCV and also as a preclinical platform to determine the effectiveness of novel vaccines and therapeutics.

**Table 5:** Antiviral treatments for HCV.

Drug name	DAA	PEG-IFNα-2A	NA808	Interferon-λ	Claudin-1
**Trade name**	Many different DAA in the market with individual names	Pegasys	-	-	-
**Manufacturing company**	A range of companies manufacture DAA	Roche Pharmaceuticals	-	-	-
**Mechanism of action**	Disrupts HCV viral life cycle by shortening the length of therapy, minimizing size effects, targeting the virus, improving sustained virological response rates	Acts as interferon within the immune system	Halts HCV replication via non-competitive inhibition of Serine Palmitoyltransferase (SPT), hence reducing viral load in mice	IFN-λ binds to heterodimeric IFN-λ receptor, activates STAT phosphorylation-dependent signal cascade which induces hundreds of IFN-stimulated genes, which in turn modulates a range of immune functions	Blocks entry of HCV
**Commonly used in combination**	PEG-IFNα-2A and Ribavirin	DAA and Ribavirin	PEG-IFNα-2A, HCV polymerase/ protease inhibitors	Not fully characterized	Ribavirin
**Rate of SVR**	~95%	~79% in genotype 1~89% in genotype 2 or 3	Not fully characterized	Not fully characterized	Not fully characterized
**Stage of clinical trial**	-	Completed and in market	-	-	-
**Side effects**	Fatigue, gastrointestinal symptoms, anemia, headache and dyspnea	Headache, fatigue, depression, insomnia, nausea, pain at site of injection, fever, psychosis, autoimmune disorders, blood clots and infection	Not fully characterized Inhibition of host enzyme might result in mechanism-related toxicities/side effects	Not fully characterized	Not fully characterized
**Advantages**	Effective Wide range of DAA	Safe Effective particularly in patients with IL28B genotype	No development of resistant mutants Able to prevent replication of HCV genotypes 1a, 1b, 2a, 3a, and 4a	Not fully characterized	Not fully characterized
**Limitations**	Expensive Unavailable in some regions of the world	Extensive and systemic side effects Limited efficacy Viral and host factors can result in non-responders	Not fully characterized	Not fully characterized	Not fully characterized
**References**	Williford et al. (2016) [3]	Huang et al. (2017) [138]	Katsume et al. (2013) [126]	Bruening et al. (2018) [139]	Colpitts et al. (2018) [140] Evans et al. (2017) [121] Meertens et al. (2008) [136]

Abbreviations - DAA: Direct-acting antiviral, HCV: Hepatitis C virus, PEG-IFNα-2A: Pegylated-interferon-α-2A, SVR: Sustained virological response, SPT: Serine Palmitoyltransferase

**Table 6:** Immunotherapy for HCC.

Drug name	Nivolumab	Pembrolizumab	Tremelimumab	Durvalumab	Ipilimumab
**Commercial name**	Opdivo	Keytruda	-	Imfinzi	Yervoy
**Company**	Bristol-Myers Squibb (BMS)	Merck Sharp & Dohme (MSD)	MedImmune	MedImmune	BMS
**Target molecule**	PD-1	PD-1	CTLA-4	PDL-1	CTLA-4
**Target cell**	T lymphocyte	T lymphocyte	T lymphocyte	Tumor cell	T lymphocyte
**Stage of clinical trial**	Approved by FDA and commercially available	Approved by FDA and commercially available	Phase III	Approved by FDA and commercially available	Approved by FDA and commercially available
**References**	El-Khoueiry AB et al. (2017) [141]	Zhu et al. (2018) [142]	Sangro et al. (2013) [143]	Wainberg et al. (2017) [144]	-

Abbreviation: BMS: Bristol-Myers Squibb, MSD: Merck Sharp & Dohme.

## 8. Immunotherapy for Hepatocellular Carcinoma

Globally, Hepatocellular carcinoma (HCC) is one of the most debilitating and fatal cancers [145]. Despite promising data from preclinical and clinical trials, current strategies for cancer treatments are limited [146]. The establishment of humanized mice has advanced knowledge of important immunopathogenesis and oncogenic signaling pathways within the diseased microenvironment of HCC [21]. In particular, HIL mice has allowed the dissection of cancer initiation and progression, as well as the opportunity to test and evaluate a diverse range of immune-oncological interventions including but not limited to, Nivolumab, Pembrolizumab, Tremelimumab, Durvalumab and Ipilimuma (Table 6).

## 9. Future direction and Conclusion

Humanized mouse models with chimeric human liver or both human immune system and hepatocytes are imperative for the characterization of HCV infection and development of therapeutics and vaccines [18,19,20,21,66,68]. As these models are of utmost importance, constant improvements are necessary to push boundaries and create models with superior clinical accuracy.

Even though current models of humanized mice are able to support HCV infection, some limitations that need to be improved on include; first, humanization levels of hepatocytes and immune cells can be further enhanced in humanized mice. This can be done through the supplementation of cytokines (IL-1β, IL-2, IL-7, and GM-CSF), to enable differentiation and maturation of HSC which can give rise to a range of immune cell subsets [147,148,149]. A more human-specific microenvironment will enable in-depth characterization of HCV immunopathogenesis and therapeutic development. Second, elimination of selective host-specific factors may improve HCV infection efficiency. For example, studies have demonstrated that very-low-density lipoprotein (VLDL) blocks HCV cell attachment; therefore, removing VLDL may increase the infection efficiency of HCV [150].

Third, the liver sinusoidal endothelium (LSEC) has a paramount role in shaping intrahepatic immune responses by mediating antigen-presentation and immune cell homing into the liver but is of mouse origin [151,152]. Finding methods to humanize this anatomy will provide valuable insights into human immune cell migration via the liver endothelium. Fourth, to fully mimic clinical settings, induced pluripotent stem cells (iPSC) may be used to create patient matched mice for HCV and HCC studies [21,153].

The lack of effective therapies and vaccine for HCV highlights an unmet clinical need. Advancements in developing humanized mouse models will provide insights into the complexity, redundancy, interdependence and regulatory mechanisms of acute and chronic HCV infection, therefore providing exciting opportunities for in vivo characterization of HCV virus-host interaction and the identification of novel vaccine and treatment strategies.

## Figures and Tables

**Table 1:** Chimeric human liver mouse models (Part I).

Name	Alb-uPA/SCID	cDNA-uPA/SCID	MUP-uPA/SCID/Bg
**Nomenclature**	-	-	-
**Engraftment method for humanization of immune system**	-	-	-
**Engraftment method for humanization of liver**	Intrasplenic injection	Intrasplenic injection	Intrasplenic injection
**Source of cells**	Human hepatocyte	Human hepatocyte	Human hepatocyte
**Presence of human hepatocytes**	Yes	Yes	Yes
**Presence of human immune system**	No	No	No
**Method of HCV infection**	Intraperitoneal injection	Intravenous injection	Intravenous injection
**Strain of HCV used**	Patient serum containing HCV genotype 1a	Serum/culture medium of HCV (10^5^ copies)	Diluted plasma from HCV-infected chimpanzee
**Duration monitored post-HCV infection**	Up to 10 weeks	Up to 8 weeks	Up to 8 weeks
**Advantages**	Presence of mature human hepatocytes This model is able to recapitulate the human immune system more efficiently than mouse models without humanization Higher human hepatocytes and HCV viraemia levels as compared to TK-NOG Useful for evaluation antiviral agents Capable of supporting long-term HCV infection	The cDNA with albumin promoter/enhancer and uPA demonstrate no loss of uPA due to the deletion of transgene Few renal disorders High body weight High survival rate Presence of mature human hepatocytes Higher hepatocyte reconstitution as compared to Alb-uPA/SCID mice Higher concentration of serum albumin as compared to Alb-uPA/SCID mice High and persistent titers of viremia Capable of supporting long-term HCV infection	Easy to maintain colony of transgenic mice High survival rate Less technically challenging to engraft hepatocytes into mice, as there is a long window to engraft mice (4-12 months) Each major HCV genotype was infectious in MUP-uPA/SCID mice Capable of supporting long-term HCV infection
**Drawbacks**	Poor breeding efficiency Short window for engraftment Absence of human immune system Liver environment is unsuitable for the engraftment of fetal-liver derived cells Unable to reproduce pathological outcomes of HCV Impossible to study HCV immunopathogenesis No liver disease Low level of hepatocyte reconstitution Low serum levels of human albumin and HCV viremia Repopulation of the liver with human cells may be cause by cell fusion No liver disease High mortality rate Low body weight High renal disorders	Absence of human immune system Unable to reproduce pathological outcomes of HCV Liver environment is unsuitable for the engraftment of fetal-liver derived cells Impossible to study HCV immunopathogenesis No liver disease	Absence of human immune system Variable viral replication observed in mice Unable to reproduce pathological outcomes of HCV Liver environment is unsuitable for the engraftment of fetal-liver derived cells Impossible to study HCV immunopathogenesis No liver disease
**References**	Washburn et al. (2011) [66] Steenbergen et al. (2010) [69]	Uchida et al. (2017) [70]	Tesfaye et al. (2017) [71] Carpentier et al. (2014) [72]

Abbreviations: cDNA: Complementary DNA, Fah: Fumarylacetoacetate hydrolase, HCV: Hepatitis C virus, NTBC: 2-(2-nitro-4-trifluoro-methylbenzoyl)1,3-cyclohexedione.

**Table 2:** Chimeric human liver mouse models (Part II).

Name	FRG	TK-NOG
**Nomenclature**	Fah^−/−^Rag2^−/−^γC^−/−^	NOD.Cg-*Prkdc^scid^ Il2rg^tm1Sug^* Tg(Alb-TK)7-2/ShiJic
**Engraftment method for humanization of immune system**	-	-
**Engraftment method for humanization of liver**	Intrahepatic injection	Intrasplenic injection of human hepatocytes
**Source of cells**	Human hepatocytes	Human hepatocytes
**Presence of human hepatocytes**	Yes	Yes
**Presence of human immune system**	No	No
**Method of HCV infection**	Intravenous injection	Intravenous injection
**Strain of HCV used**	Supernatant of Huh-7 cell culture containing 2 × 10^4^ ffu JFH-1, 3 × 10^3^ ffu HCV Con1/C3, 3 × 10^3^ ffu HCV H77/C3 Patient serum containing 2 × 10^5^ GE/ml HCV genotype 1a Patient serum containing 2 × 10^5^ IU/ml HCV genotype 3a	Patient serum containing HCV genotype 1b (2.2 x 10^6^ copies/mL)
**Duration monitored post-HCV infection**	Up to 5 weeks	Up to 8 weeks
**Advantages**	Simplified animal husbandry and surgery as liver disease can be controlled by NTBC Mice are genetically stable Pharmacological interference not needed in reconstituting FRG mice with hepatocytes Can be serially engrafted with human hepatocytes No renal disorders High survival rate Capable of supporting long-term HCV infection	Able to achieve endogenous liver injury and human hepatocyte engraftment Cost effective as compared to uPA/SCID and FRG mice Capable of supporting long-term HCV infection
**Drawbacks**	Requires maintenance under constant and costly NTBC treatment Absence of human immune system Primary engraftment does not occur in all recipient mice Able to achieve high human hepatocyte reconstitution with only human adult liver cells Unable to reproduce pathological outcomes of HCV	Absence of human immune system Liver environment is unsuitable for the engraftment of fetal-liver derived cells Unable to reproduce pathological outcomes of HCV High mortality rate Low body weight High renal disorders
**References**	Washburn et al. (2011) [66] Azuma et al. (2007) [73] Bissig et al. (2010) [74]	Dagur et al. (2018) [75] Kosaka et al. (2013) [76]

**Table 3:** Humanized mouse models with both human immune system and hepatocytes.

Name	AFC8-hu HSC/Hep	NSG-DRB*0101
**Nomenclature**	AFC8-HSC/Hep Balb/C Rag2^−/−^γC^−/−^	NOD/scid-DRB*0101
**Engraftment method for humanization of immune system**	Intrahepatic injection	Intrahepatic injection
**Engraftment method for humanization of liver**	Intrahepatic injection	Intrahepatic injection
**Source of cells**	Human adult cells	Human fetal liver
**Presence of human hepatocytes**	Yes	Yes
**Presence of human immune system**	Yes	Yes
**Method of HCV infection**	Intravenous injection	Intravenous injection
**Strain of HCV used**	Clinical isolate of HCV genotype 1a (1-5 x 10^7^ genome copies/mL)	Recombinant adenovirus serotype 5 (AdV5) (5 x 10^9^ or 10^10^ particles)
**Duration monitored post-HCV infection**	Up to 20 weeks	Up to 4 weeks
**Advantages**	The use of caspase 8-dependent induction of mouse hepatocyte apoptosis to promote human hepatocyte repopulation is less toxic as compared with uPA/SCID and FAH mice Presence of both human immune system and hepatocytes AFC8-hu HSC/Hep mice infected with HCV generates human immune responses, elevated levels of alanine aminotransferase (ALT), liver inflammation, hepatitis and fibrosis Suitable for the study of hepatitis virus-induced liver immunopathogenesis HCV genomic RNA is detectable in the livers of mice Only small animal model capable to support the co-infection of HCV and HIV Useful platform for the evaluation of antiviral drugs and immunotherapies	Presence of both human immune system and hepatocytes Transgenic HLA expression improves human antiviral HLA-restricted T cell responses during human viral infections Suitable for the study of hepatitis virus-induced liver immunopathogenesis
**Drawbacks**	Liver sinusoidal endothelium is of mouse origin Low level of repopulation and immature phenotype of human hepatocytes Unable to detect significant HCV viremia in the blood Low serum levels of human albumin and HCV viremia Cannot be used for long-term studies Antiviral immune responses may not be as robust as in human patients Does not fully recapitulate clinical settings	To analyze HCV immunopathogenesis, mice must be engrafted with both donor matched human hepatocytes and immune cells, hence making this a challenging model to establish Liver sinusoidal endothelium is of mouse origin Lack of complete viral clearance from the liver Does not fully recapitulate clinical settings
**References**	Washburn et al. (2011) [66] Bility et al. (2012) [67]	Billerbeck et al. (2013) [68]

**Table 4:** Humanized mouse models with both human immune system and hepatocytes (HIL mice).

Name	NSG	NSG	NSG
**Nomenclature**	NOD-scid Il2rg^−/−^	NOD-scid Il2rg^−/−^	NOD-scid Il2rg^−/−^
**Engraftment method for humanization of immune system**	Intrahepatic injection	Intrahepatic injection	Intrahepatic injection
**Engraftment method for humanization of liver**	Intrahepatic injection	Intrahepatic injection	Intrahepatic injection
**Source of cells**	HLA type I matched fetal liver	Fetal liver	Fetal liver
**Presence of human hepatocytes**	Yes	Yes	Yes
**Presence of human immune system**	Yes	Yes	Yes
**Method of HCV infection**	Intravenous	Intravenous	Intravenous
**Strain of HCV used**	HCV induced HCC patient derived xenograft	10^6^ FFU/mL of J6/JFH-1 HCV (genotype 2a) viruses	10^6^ or 10^7^ ffu of HCVJ6/JFH1-P47
**Duration monitored post-HCV infection**	Up to 8 weeks	Up to 9 weeks	Up to 28 weeks
**Advantages**	Presence of both human immune system and hepatocytes Liver inflammation and fibrosis are observed Able to study HCV immunopathogenesis Useful platform for therapeutic testing Side effects of immunotherapies tested on this mouse model were similar to clinical settings	Presence of both human immune system and hepatocytes Liver inflammation and fibrosis are observed Mice are able to support HCV infection and demonstrate some clinical symptoms found in HCV-infected patients including hepatitis, robust virus-specific human immune cell and cytokine response as well as liver fibrosis and cirrhosis Useful platform for therapeutic testing such as antiviral treatment, PEG-IFNα-2A	Presence of both human immune system and hepatocytes HCV infected mice developed increase incidences of liver fibrosis, granulomatous inflammation and tumour formation in the form of hepatocellular adenomas/carcinomas by 28-weeks post-infection as compared to uninfected mice Mice can recapitulate some clinical symptoms, such as, chronic inflammation, immune cell exhaustion and tumorigenesis as observed in patients HCV infection is able to progress beyond 27-weeks in this model Liver inflammation and fibrosis are observed Able to study HCV immunopathogenesis Useful platform for therapeutic testing
**Drawbacks**	Does not fully recapitulate HCV responses as observed in patients Antiviral immune responses not as robust as observed in clinical settings	Does not fully recapitulate HCV responses as observed in patients Antiviral immune responses not as robust as observed in clinical settings	Does not fully recapitulate HCV responses as observed in patients Antiviral immune responses not as robust as observed in clinical settings Effects of HCV in mice needs to be monitored for a longer duration of time to confirm if liver tumorigenesis can occur Despite observation of chronic hepatitis, viremia was not detectable in plasma/liver of infected mice HCV RNA was challenging to detect with non-sacrificial sampling methods HCV RNA could only be detected after extracting RNA from purified human hepatocytes in infected HIL mice, although this renders the liver tissue unusable for histopathological analyses
**References**	Zhao et al. (2018) [21]	Keng et al. (2015) [18]	Zheng et al. (2017) [19]
